# COVID-19 Presenting as Recurrent Pericardial Effusion

**DOI:** 10.7759/cureus.18652

**Published:** 2021-10-11

**Authors:** Dena H Tran, Anuj Gupta, Avelino C Verceles, Robert D Chow

**Affiliations:** 1 Internal Medicine, University of Maryland Medical Center Midtown Campus, Baltimore, USA; 2 Cardiovascular Medicine, University of Maryland School of Medicine, Baltimore, USA; 3 Pulmonary and Critical Care Medicine, University of Maryland School of Medicine, Baltimore, USA

**Keywords:** pericardial effusion, covid and heart, pericardial window, sars-cov-2 (severe acute respiratory syndrome coronavirus -2), covid-19

## Abstract

Severe acute respiratory syndrome coronavirus (SARS-CoV-2) emerged from Wuhan, China, in 2019, causing coronavirus disease 19 (COVID-19) and creating a global pandemic affecting millions of people worldwide. Though COVID-19 primarily affects the pulmonary structures, deleterious effects can also occur in the cardiac system. We present a case of a patient with recurrent pericardial effusions secondary to COVID-19 infection, an unusual cardiovascular manifestation of this disease. A 47-year-old man presented with altered mental status and tested positive for COVID-19. He left against medical advice and later presented two weeks later with pleuritic chest pain associated with shortness of breath. His symptoms were attributed to a moderate- to large-sized pericardial effusion, without evidence of tamponade, as confirmed by echocardiography. The fluid was removed by pericardiocentesis; analysis was negative for malignant cells, inflammatory markers, or microbiologic studies. Reaccumulation of the fluid necessitated placement of a pericardial window, resulting in the resolution of his symptoms. There are limited case reports demonstrating the association of pericardial effusion with COVID-19 infection. The effusion is likely secondary to the inflammatory response leading to capillary leakage, resulting in pericardial fluid traversing the serous pericardium. In addition to other demonstrated cardiovascular effects, COVID-19 appears to be associated with recurrent pericardial effusion. Due to the rise in COVID-19 cases, it is essential to consider pericardial effusion as a rare but potential complication of this virus. The pericardial effusion can be the primary clinical manifestation, recurrent in nature, and potentially result in tamponade physiology.

## Introduction

Severe acute respiratory syndrome coronavirus (SARS-CoV-2) emerged from Wuhan, China, in 2019, resulting in coronavirus disease 19 (COVID-19) and a global pandemic that affected millions of people worldwide. As of early September 2021, over 219 million total COVID-19 cases have been reported with 4.5 million deaths [1]. COVID-19 primarily causes respiratory illness, including fever, cough, myalgia, fatigue, shortness of breath, and even acute respiratory distress syndrome [2]. Deleterious effects on the cardiovascular system, particularly the myocardium, have been demonstrated [3]. Acute cardiac injury, acute coronary syndrome, thrombosis, and pulmonary embolism have all been associated with COVID-19 infection. However, there have been few documented cases focusing on COVID-19 causing pericardial effusions. We present a case of a patient who presented with recurrent pericardial effusions likely secondary to COVID-19 infection.

## Case presentation

A 47-year-old man presented to the emergency department (ED) in April 2021 for altered mental status. His medical history consisted of type 2 diabetes mellitus and hypertension, and there was no history of malignancy. Per Emergency Medical Services, the patient lives at home with a roommate who witnessed a seizure-like episode. He arrived at the ED with glucose in the 700s. Home medications included insulin glargine 25 units twice daily, gabapentin 800 mg three times daily, aspirin 81 mg daily, atorvastatin 40 mg daily, and metoprolol succinate 200 mg daily. He was treated for hyperosmolar hyperglycemic state in the ED and was admitted for further management. The patient was found to be COVID positive on SARS-CoV-2 RNA testing. He left against medical advice two days later. 

**Figure 1:**
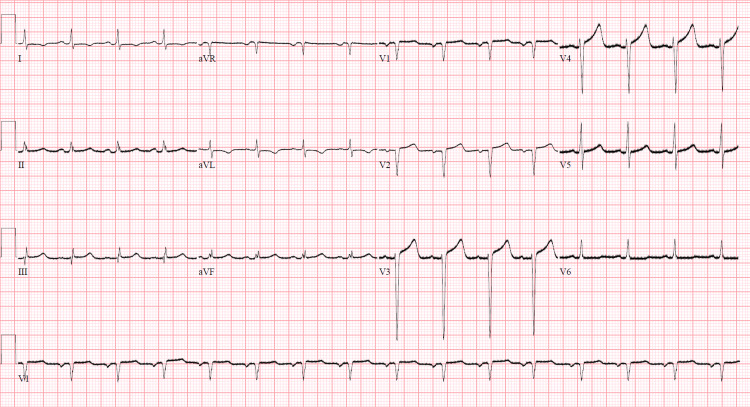
Electrocardiography Normal sinus rhythm with low voltages and an isolated Q wave in lead III without diffuse ST-segment elevations. aVR: augmented vector right, aVL: augmented vector left, aVF: augmented vector foot.

Approximately two weeks later, the patient presented with pleuritic chest pain associated with shortness of breath. Vital signs at the time of admission: temperature 37.9° Celsius, heart rate 92 beats/minute, blood pressure 117/79 mmHg, respiratory rate 18 breaths/minute, and oxygen saturation 94% on room air. Cardiac examination revealed normal S1 and S2 and no murmurs, rubs, or gallops. Lung auscultation was clear to auscultation bilaterally, without wheezes, rhonchi, or rales. Bilateral 2+ pitting edema was present to the level of his knees. Initial troponin was 0.481 ng/ml (reference <0.012 ng/ml) but subsequently downtrended to <0.02 ng/ml, and pro B-type natriuretic peptide (pro-BNP) was 2800 pg/ml (reference 0-300 pg/ml). Electrocardiography (ECG) showed normal sinus rhythm with low voltages in the limb leads; there were no diffuse ST-segment elevations (Figure 1). Transthoracic echocardiogram (TTE) showed a left ventricular ejection fraction of 30% with severe global hypokinesis, increased wall thickness of the right ventricle, and a small circumferential pericardial effusion without evidence of tamponade physiology. Unfortunately, the patient again left against medical advice.

Three months later, the patient presented to the hospital with shortness of breath and was found to be volume overloaded due to medication noncompliance. TTE showed a large pericardial effusion with diastolic compression of the right atrium, consistent with cardiac tamponade physiology. He underwent pericardiocentesis with the removal of 800 ml serosanguinous fluid that was negative for malignant cytology. Acid-fast bacilli culture and smear of the pericardial fluid were negative. Approximately one month later, the patient saw his cardiologist for follow-up. The patient was referred to the ED for bilateral lower extremity cellulitis. On this most recent ED visit, approximately four months after the initial presentation, the patient agreed to stay in the hospital and undergo a comprehensive diagnostic evaluation.

**Figure 2:**
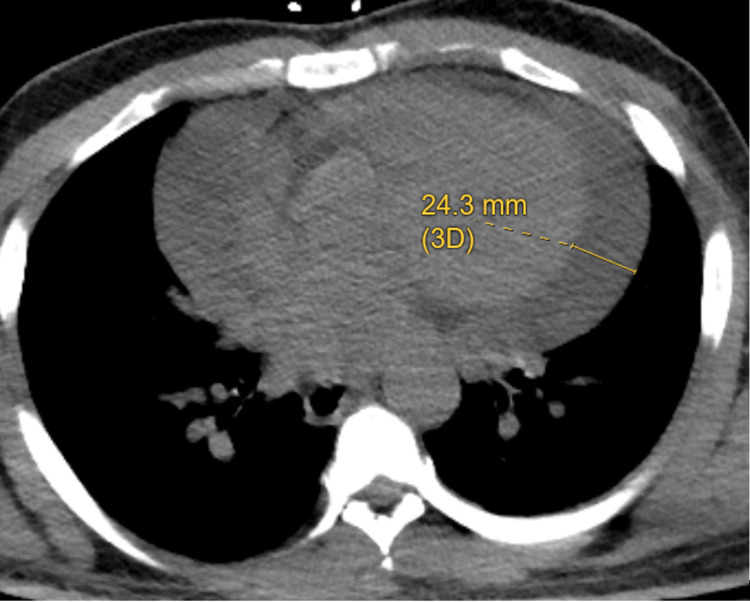
Computed Tomography Chest Pericardial effusion of 24.3 mm (3D) shown on computed tomography chest imaging. D: dimension.

In addition to evaluation of his cellulitis, bedside ultrasonography showed that a moderate to large pericardial effusion had recurred, though without evidence of right ventricular diastolic collapse or right atrial systolic collapse. CT chest also confirmed an incidental large pericardial effusion (Figure 2). Blood cultures were negative. Testing of human immunodeficiency virus (HIV) and antinuclear antibody (ANA) serology was negative. His ECG on this admission was unchanged from prior ECGs. Hemoglobin A1c was 8%, and low-density lipoprotein (LDL) was 58 mg/dl. Carcinoembryonic antigen (CEA), alpha fetoprotein (AFP), cancer antigens (CA 19-9), and prostate-specific antigen (PSA) were negative. Repeat TTE showed large pericardial effusion (2.3 cm) and evidence of tamponade physiology (exaggerated respiratory variation in mitral inflow velocity (E) on Doppler echocardiography). 

Of note, a previous left heart catheterization with coronary angiography did not show significant obstructive coronary artery disease. A prior echocardiogram seven months prior revealed an ejection fraction of 65%, without pericardial effusion. The patient underwent placement of a pericardial window, and a chest tube was inserted. Repeat blood cultures were negative. The chest tube drained approximately 165 ml. The patient’s clinical presentation improved, and he was discharged home.

## Discussion

COVID-19 can cause pericardial effusions in patients with myocarditis/pericarditis [4]. The proposed mechanism of action involves both the direct involvement of the SARS-CoV-2 virus and its indirect effects through the cytokine storm and oxidative stress reaction. SARS-CoV-2 binds to the angiotensin-converting enzyme 2 (ACE2) to activate the ACE2 signaling pathway, which induces myocardial injury leading to pericardial effusion. It is also thought that the release of the cytokine storm, and in particular the inflammatory agents tumor necrosis factor-alpha (TNF-alpha), interleukins (IL-1, IL-6, and IL-8), may result in myocarditis and perimyocarditis [5]. The pericardial effusion may be secondary to a capillary leakage phenomenon, in which pericardial fluid is able to traverse the serous pericardium.

Amoozgar et al. have similarly reported a case of pericardial effusion in the setting of asymptomatic COVID-19 infection for which the patient received a pericardial window followed by ibuprofen and then was successfully discharged home [6]. Sauer et al. reported three cases of pericardial effusions secondary to COVID-19 with varying ranges of cardiac tamponade, where the patients were treated with colchicine, and their conditions had rapidly improved [7].

After infection with COVID-19, our patient developed recurrent pericardial effusions. Prior to his COVID-19 infection, he did not have evidence of pericardial effusion on TTE. The infectious workup of the pericardial effusion fluid was sterile, and his autoimmune and malignancy workup were negative. Thus, his pericardial effusion was most likely due to COVID-19 infection. 

## Conclusions

Though the clinical manifestations of COVID-19 are primarily pulmonary in nature, cardiac complications can arise in the absence of pulmonary findings. There have been limited studies documenting COVID-19 viral infection as a cause of pericardial effusions. We present a case of a patient who had recurrent episodes of pericardial effusion due to COVID-19 infection.
